# Short Version Dental Anxiety Inventory Score May Predict the Response in the Insular Cortex to Stimuli Mimicking Dental Treatment

**DOI:** 10.3389/fnhum.2019.00204

**Published:** 2019-06-11

**Authors:** Andy Wai Kan Yeung, Johnson Chun Ming Lee, Hiroki C. Tanabe, Sam Kwai Sang Ng, Pek-Lan Khong, Wai Keung Leung, Tazuko K. Goto

**Affiliations:** ^1^Oral and Maxillofacial Radiology, Applied Oral Sciences, Faculty of Dentistry, The University of Hong Kong, Hong Kong, China; ^2^Private Practice, Hong Kong, China; ^3^Department of Psychology, Graduate School of Environmental Studies, Nagoya University, Nagoya, Japan; ^4^Periodontology, Faculty of Dentistry, The University of Hong Kong, Hong Kong, China; ^5^Department of Diagnostic Radiology, Li Ka Shing Faculty of Medicine, The University of Hong Kong, Hong Kong, China; ^6^Department of Oral and Maxillofacial Radiology, Tokyo Dental College, Tokyo, Japan

**Keywords:** dental anxiety, dental equipment, emotions, functional neuroimaging, neurosciences

## Abstract

**Background**: Dental anxiety is a common reason for avoiding dental visits and is associated with poor dental status. The short version of Dental Anxiety Inventory (SDAxI) is an easy-to-use, multi-faceted questionnaire for assessing the level of trait dental anxiety. However, there was no neurophysiological data indicating if its score associates with the state anxiety when an individual is under real/mock dental environment. We hypothesized that there exists such an association.

**Materials and Methods**: Twenty systemic healthy adults with dental attendance experience and self-claimed free of dental phobia were recruited in this cross-sectional study, with their dental anxiety level assessed by SDAxI. Functional magnetic resonance imaging recorded their brain signals in response to audiovisual footages resembling dental scaler or turbine in action. After the brain imaging, they gave fear ratings to the footages in visual analog scale (VAS).

**Results**: Participants’ SDAxI scores positively correlated with their responses in the insular cortex (*r^2^* = 0.388–0.445, *P* < 0.005). Their SDAxI scores also positively correlated with their fear ratings of the footages (*r*^2^ = 0.415–0.555, *P* < 0.005).

**Discussion**: Our findings indicated a possible neurobiological relevance of SDAxI, and reinforced its neurobiological validity in assessing dental anxiety level of dental attenders.

## Introduction

There are many dental therapy scenarios and dental treatment settings that make patients feel threatened and anxious about (Oosterink et al., 2008). Dental anxiety is a commonly cited reason for avoidance or postponement of dental visits by patients and is associated with poor dental status and reduced oral health-related quality of life (McGrath and Bedi, 2004; Ng and Leung, 2008). Individual with higher dental anxiety could choose to refuse appropriate intervention such as local anesthesia thus rendering dental treatment an uncomfortable and less satisfying experience (Leung et al., 2016). The psychosocial research field has designed and tested scales to assess dental anxiety and identify potentially anxious dental patients (Stouthard et al., 1993, 1995; Aartman, 1998; Armfield, 2010a). On the other hand, to understand the neural basis of dental anxiety, the neurophysiology research field has employed neuroimaging techniques, such as functional magentic resonance imaging (fMRI), to observe brain responses triggered by stimuli mimicking dental treatment or scenarios with dental anxiety context (Lueken et al., 2011; Hilbert et al., 2014; Scharmüller et al., 2014; Schienle et al., 2014; Yeung et al., 2019). When such research findings from both fields are translated to clinical and educational purposes, clinicians can improve their delivery of care and dental patients will be greatly benefited (Yeung et al., 2017). In short, dental anxiety hinders patients from having regular dental visits, which are essential for maintaining good oral health and quality of life. Therefore, dental anxiety is an important issue to be tackled as it bears significant impact on oral health-related quality of life, and that general anxiety may not be a good reflection of it.

The easy-to-use, multi-faceted short version of Dental Anxiety Inventory (SDAxI) has been extensively employed to profile the psychosocial dental anxiety level of dental patients and the general population (Stouthard et al., 1993; Ng et al., 2005; de Jongh et al., 2008, 2011; Ng and Leung, 2008; Vermaire et al., 2008; Oosterink et al., 2009; van Wijk and Hoogstraten, 2009; Lindeboom and van Wijk, 2010; van Wijk et al., 2010; Ikeda and Ayuse, 2013). Its application is not limited to a small number of special cases in dental clinic with patients suffering from dental phobia; instead, it has a broad application to patients coming from general population. However, no relevant neuroimaging data has been published to further evaluate or support the use of SDAxI.

Various efforts have been made to connect the neurophysiological and psychosocial findings on dental anxiety, and results have been implying that the insula in the brain is strongly related to processing of dental anxiety. For instance, efforts have been made to correlate brain responses in the insula with some anxiety assessment scores other than SDAxI, but results were significant either with tools not specific for dental anxiety or among specific target groups only. For instance, a study by Schienle et al. (2014) revealed a positive correlation between the brain response in the insula and the trait score of the State-Trait Anxiety Inventory among healthy controls who were watching dental treatment photographs. However, trait score of the State-Trait Anxiety Inventory is a scale designed to assess general anxiety but not dental anxiety (Taylor and Deane, 2002). With the same photographs forming the basis of the stimuli, their research group also found the response in the insula was larger from a dental phobic patient group with high Corah’s Dental Anxiety Scale as compared to a healthy control group (Schienle et al., 2013). Also, there was a positive correlation between the response in the insula and Corah’s Dental Anxiety Scale score, but the correlation existed among female dental phobics only (Scharmüller et al., 2014). On the other hand, Lin et al. (2015) revealed a positive correlation of brain response in the insula with a customized, generic dental avoidance score among healthy participants who were watching dental treatment photographs. However, the validation of this inventory has not been published. At the same time, Hilbert et al. (2014) used Dental Fear Survey to recruit subjects and found the dental phobic group had larger responses in the insula than the healthy control group when they listened to sounds from dental drills. All these findings support the role of insula in processing stimuli mimicking dental treatment or dental anxiety, and it may be possible to characterize the brain responses of general or specific participants exposed to stimuli mimicking dental treatment, and hence correlate and verify the applicability of the psychosocial tool concerned.

To the best of our knowledge, however, no published studies have reported a correlation between the “neurophysiological” brain responses subjected to stimuli mimicking dental treatment in the insula and the “psychosocial” scores from a validated, standardized trait dental anxiety scale among self-perceived healthy, non-dental phobic individuals. We think the general population, as represented by healthy adults, is important because they represent the majority and most of them will also visit a dentist for checkup or treatment. If someone with dental anxiety receives the recommended dental care, they will feel more comfortable and have a better dental treatment experience if we know the biology behind and ways to alleviate the anxiety. Therefore, the aim of this study was to investigate if there is any association between SDAxI scores and the brain responses of healthy adults recorded by fMRI to standardized dental scaler and turbine videos. Participants would rate the scariness of videos using visual analog scale (VAS). We hypothesized SDAxI and the perceived scariness of the mock stimuli would positively correlate with the level of brain activity in the insula among participants who self-reported with no dental phobia.

## Materials and Methods

### Participants

The association between SDAxI and brain response has not been investigated before. However, considering evidence from previously published reports that focused on the correlation between brain response and behavioral score (e.g., score assessing dental anxiety or dental avoidance) with a similar hypothesized model as employed in this study, a sample size of 19 would be needed (Mumford and Nichols, 2008; Scharmüller et al., 2014; Lin et al., 2015). We therefore planned to recruit 20 healthy adults (10 women, 10 men) for the current study. Participants were recruited from The University of Hong Kong community by means of advertisements or introduction by persons previously participated in experiments from our laboratory. They must meet all of the following inclusion criteria: (1) could undergo an MRI scan of the head; (2) had prior experience involving receiving dental treatment by dental scaler and turbine (handpiece) intra-orally without wearing eye masks or earplugs and thus could recognize the sight of the instruments; (3) understood the function of the scaler and turbine and could distinguish between them without hint/cue when they see their pictures; and (4) had not been clinically diagnosed or self-reported with dental phobia. Participants were excluded if they meet any of the following criteria: (1) could not undergo MRI (because of claustrophobia, back pain, or possession of MRI-contraindicated metal devices such as a pacemaker); (2) were currently taking any medication; (3) had active or past history of neurological or psychiatric illnesses; and (4) currently had pain or discomfort related to dental problem, or claimed they currently had uncontrolled active dental diseases. After explaining all study procedures, participants completed an SDAxI questionnaire (Ng et al., 2005). The questionnaire consisted of nine questions, to be answered on a 5-point Likert scale, ranging from 1, “completely disagree” to 5, “completely agree.” A final score was computed by tallying the scores of the items, with the total score ranging from 9 to 45. The original SDAxI questionnaire in English version was developed and published by Aartman (1998). The advantage of SDAxI was a tool considered to be different from Dental Anxiety Scale, as the response categories of the latter does not cover the multiple facets of dental anxiety as SDAxI does. SDAxI is also different from Dental Fear Survey, which was not originally developed as a measure of dental anxiety so that it evaluated some irrelevant items such as dental avoidance and hence the summated score cannot accurately reflect dental anxiety level (Armfield, 2010b). As elaborated in the “Introduction” section, SDAxI profiles the trait dental anxiety level of the participants. Similar to Lin et al. (2015), we did not recruit participants who claimed to have dental phobia because we aimed at revealing neural correlates generalizable to the majority of population. The Institutional Review Board of The University of Hong Kong/Hospital Authority Hong Kong West Cluster approved the study (IRB UW 11-191). The study was carried out in accordance with the World Medical Association Declaration of Helsinki (version 2002) and written consent was obtained from all participants.

### Imaging Data Acquisition

The fMRI scanning was performed in a 3-Tesla scanner (Philips Achieva 3.0 System; Philips Medical System, Netherlands) in the 3T MRI Unit, Department of Diagnostic Radiology, The University of Hong Kong. We reduced movement artifacts by securing the participants’ heads with straps and small, wedge-shaped pillows. Functional images were obtained using a T2*-weighted gradient-echo planar imaging (EPI) technique (repetition time or TR = 2 s, echo time or TE = 30 ms, flip angle = 80°, field-of-view or FOV = 220 mm, matrix size = 80 pixels × 80 pixels, voxel size = 2.75 mm × 2.75 mm × 3.5 mm, slice thickness = 3.0 mm, slice gap = 0.5 mm). Each volume of data consisted of 31 slices to cover the whole brain, and a 330-volume data set was acquired in each of two successive 11-min scanning sessions. Two sessions were arranged instead of one due to limited number of scans acquirable by the MRI machine. Scaler and turbine videos were shown in pseudo-randomized sequence.

To obtain anatomical scans for each subject, we acquired T1-weighted MPRAGE images (TR = 6,935 ms, TE = 3,129 ms, flip angle = 8°, FOV = 250 mm, matrix size = 256 pixels × 256 pixels, voxel size = 0.977 mm × 0.977 mm × 1 mm, slice thickness = 1.0 mm), with the same location variables as those of the EPI (Goto et al., 2016; Yeung et al., 2016). We did not segment the T1-weighted images into GM and WM. For each participant, we co-registered the T1 image to their functional scans during data preprocessing. This aligned the structural and functional images into the common anatomical space, so that we could study where the activations were, in terms of the participant’s own anatomy.

### fMRI Protocol

**Figure 1:**
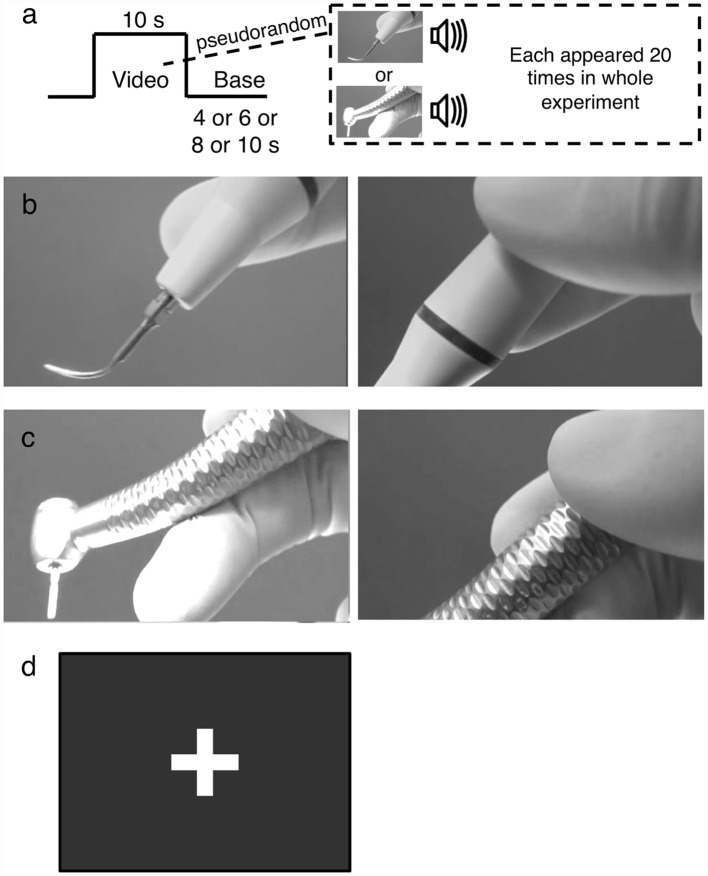
The experimental design. **(A)** Example of the functional magentic resonance imaging (fMRI) block paradigm. Audiovisual footages of either a scaler or turbine were shown 20 times for 10 s each in a pseudorandomized order, alternating with a base image. The base image was pseudorandomly selected to be shown for 4, 6, 8, or 10 s. **(B)** The scaler video. It initially focused on the scaler tip (left panel) and zoomed in on the handle (right panel). **(C)** The turbine video. It initially focused on the drill attachment (left panel) and zoomed in on the handle (right panel). **(D)** The base. It was a still image of a white cross on a black background without any audio signal incorporated.

To elicit brain activities associated with stimuli mimicking dental treatment, audiovisual footages of scaler and turbine were used as stimuli. Footages were employed because audiovisual footage of stimuli mimicking dental treatment could effectively activate the insula (Hilbert et al., 2014) and are more realistic compared to static, silent photographs or sound alone. Scaler and turbine were chosen because they are routinely used and rank high in the hierarchy of conditions that provoke dental anxiety (Gale, 1972; Stouthard and Hoogstraten, 1987; McNeil et al., 1993; Wong et al., 2011, 2015). Each stimulus was shown 20 times in the whole experiment (Figure 1A). The scaler (Figure 1B) and turbine (Figure 1C) were shown as being held in a gloved hand. The video started at the tip and zoomed in on the handle as the scaler and turbine were lowered, while the corresponding sound tracks were produced by operating the equipment on a plastic tooth behind the scenes, to simulate what patients might see and hear intraoperatively. Both videos were matched for their foreground-background ratios, speed of zooming, and sound level (Lueken et al., 2011; Hilbert et al., 2014). The delivery of the videos was controlled by E-prime software and the timing to show each audiovisual stimulus was synchronized with the acquisition of fMRI scans.

The images were relayed from a screen in the 3T MRI scanner room to a mirror above the participants’ heads. The participants wore a headphone to receive the audio stimuli, and were instructed to concentrate on sensing the audiovisual footages without an explicit cognitive task. A block design was used to play the videos in a pseudorandomized order in alternation with a base image of a white cross on a black background (Figure 1D). The duration of each stimulus video was 10 s and that of the base image was pseudorandomly selected as being 4, 6, 8, or 10 s. The brain responses to the audiovisual footages were recorded by the MRI machine.

### Video Evaluation

Immediately after MRI scanning, each participant rated the videos on a 0–100 mm VAS regarding their perceived dental fear level with “0” representing no fear at all to “100” being extremely fearful. This VAS rating showed how fearful a participant was during the audiovisual stimulation inside the MRI scanner, and such measurement has been used in previous publication to evaluate level of fear (Vlaeyen et al., 1995). This VAS rating was used to correlate with the level of brain responses.

### fMRI Data Preprocessing

The fMRI data were analyzed with Statistical Parametric Mapping (SPM) 8 (Wellcome Trust Centre for Neuroimaging, UCL, London, UK) implemented in Matlab (MathWorks, Natick, MA, USA). The first four scans of each session were discarded to allow both the magnetic field and the subject to reach a steady state. Spatial preprocessing consisted of realigning functional images for motion correction, coregistering the structural image to the functional images, and normalizing both functional and anatomical images into a brain template (Montréal Neurological Institute, Montréal, QC, Canada). Functional images were then subsampled to a voxel size of 3 mm × 3 mm × 3 mm and smoothed using a Gaussian kernel with full width at half-maximum of 8 mm. Spatial smoothing was performed to improve the signal detection power (Strother, 2006).

### fMRI Data Analysis

A general linear model was applied to the fMRI data from each individual. The head motion parameters estimated from the realignment procedure were included to remove any effect caused by head movements. No subject had large head motion of >3 mm translation. Two regressors (scaler, turbine) were modeled. These regressors were convolved with a canonical hemodynamic response kernel. The video periods were designated as 10-s boxcar functions. The base image period was set as the baseline (Figure 1A). The time series data for each voxel were high-pass filtered with a cut-off period of 128 s to remove low-frequency signal drift. A first-order autoregressive model or AR(1), was used to remove serial correlations in the data. The data analysis was performed by measuring the video—baseline difference as a defined linear contrast (Worsley, 2001). Scaler and turbine videos were modeled as contrasts separately because they might elicit dental anxiety differently; the former is for cleaning and the latter is for drilling. For each contrast, a collection of *t-*statistic values from every voxel produced a statistical parametric map, which was transformed into the unit normal distribution (SPM *z*). The data was assessed at the group level with random-effects analysis to allow for population inferences. The contrasts from each subject were entered into two independent group analyses, where SDAxI scores given by the corresponding subjects were entered as a regressor into one analysis and dental fear VAS scores into another. In this way, we could identify which voxels had their activities significantly correlated with SDAxI score, or with dental fear VAS scores, respectively. Voxels significantly correlated with SDAxI had their level of activity (i.e., how intense the signal was coming from a particular voxel in response to the stimulations, also can be interpreted as effect size and known as contrast estimate in the SPM software used for data analysis) extracted and plotted with SDAxI to illustrate the linear correlation quantified by coefficient of determination *r*^2^.

We examined the whole brain for voxels with significant correlations, with the statistical threshold set at an uncorrected peak *P* value of < 0.005 and a cluster size of >10 voxels, which produces a desirable balance between Type I and II error rates (Forman et al., 1995; Lieberman and Cunningham, 2009) and is a common standard for correlation of brain data with behavioral data (Eisenberger et al., 2003; Burklund et al., 2007).

### Statistical Analyses on Behavioral Data

SPSS 20.0 (IBM, New York, NY, USA) was used to perform statistical analyses. Two-sample *t*-test was used to evaluate if there were differences between mean VAS scores of scaler and turbine. To investigate the correlation between SDAxI scores and the dental fear VAS scores for either video, Pearson’s correlation tests were carried out. Test results with a *P* value < 0.05 were considered statistically significant.

## Results

### Demographic Background of the Participants

The mean age of the participants was 27.0 years (Table 1). All participants were right-handed. There were 17 Chinese, two Japanese and one Malaysian. For non-Chinese participants, the English version of SDAxI was used. Most of the participants were studying in undergraduate programs.

**Table 1:** Demographic information of the subjects.

		Male (*n* = 10)	Female (*n* = 10)	Total	*P* value
Age (years)	(mean ± SD)	21.6 ± 2.6	32.3 ± 14.2	27.0 ± 11.3	0.042^b^
	(range)	18–27	18–61	18–61	
SDAxI^a^	(mean ± SD)	20.0 ± 10.5	24.4 ± 8.8	22.3 ± 9.7	0.334^b^
	(range)	9–44	16–42	9–44	
Dental fear VAS (mm)					
Scaler	(mean ± SD)	25.2 ± 16.3	46.1 ± 29.8	35.6 ± 25.7	0.072^b^
	(range)	0–61.0	4.8–90.0	0–90.0	
Turbine	(mean ± SD)	27.5 ± 23.3	48.1 ± 29.9	37.8 ± 28.2	0.104^b^
	(range)	4.0–76.5	0.9–90.0	0.9–90.0	
Education (n)	Secondary school	7	8	15	0.606^c^
	University degree holder	3	2	5	

*VAS, visual analog scale (0–100 mm). ^a^Levels of dental anxiety: low, score <10.03; average, score between 10.03 and 21.3; high, score >21.3 (Ng and Leung, 2008). ^b^Two-sample *t*-tests. ^c^Chi-squared test*.

### SDAxI Score

The mean SDAxI score of the study participants was 22.3 (Table 1). Using the classification by Ng and Leung (2008), this number stands in the middle of average to high dental anxiety.

### Dental Fear VAS Scores

The mean VAS scores of the study participants were 35.6 mm for the scaler video and 37.8 mm for the turbine video. There was no significant difference between males and females (Table 1), and between mean VAS scores of scaler and turbine (*P* = 0.799, two-sample *t*-test).

### Correlation Between Brain Activities and SDAxI Score

**Figure 2:**
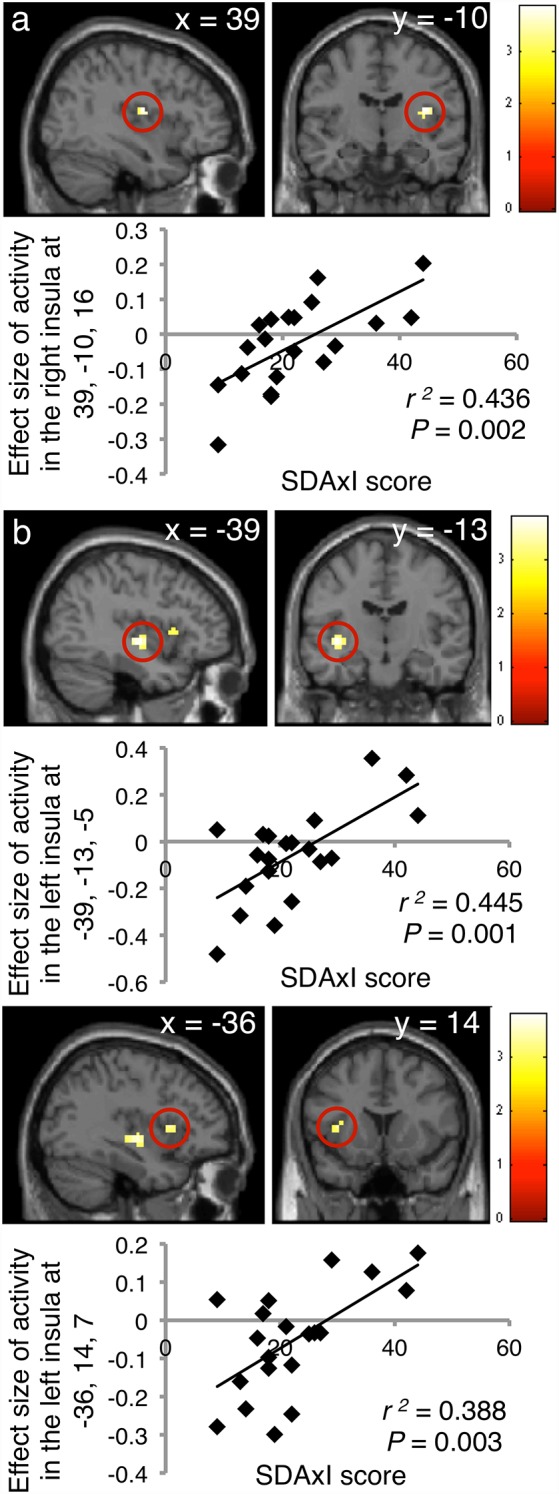
Associations of brain responses to audiovisual footages of dental scaler or turbine with short version of Dental Anxiety Inventory (SDAxI) score (*n* = 20).** (A)** Scaler video. Responses in the right insula were positively correlated with the SDAxI score. **(B)** Turbine video. Responses in the left insula (first peak, upper panel; second peak, lower panel) were positively correlated with the SDAxI score.

Activities in the insula triggered by both the scaler and turbine audiovisual footages correlated positively with the subject SDAxI scores (Table 2). For the scaler video, there was a positive correlation between brain response in the right insula (peak voxel at 39, −10, 16, cluster size = 21 voxels, *z* = 3.17) and the subject SDAxI score (*r*^2^ = 0.436, *P* = 0.002; Figure 2A). For the turbine video, there was a positive correlation between brain responses in the left insula (peak voxel at −39, −13, −5, cluster size = 41 voxels, *z* = 3.21; peak voxel at −36, 14, 7, cluster size = 14 voxels, *z* = 2.93) and the subject SDAxI scores (*r*^2^ = 0.445, *P* = 0.001 and *r*^2^ = 0.388, *P* = 0.003, respectively; Figure 2B).

**Table 2:** Brain regions with level of activity in response to scaler or turbine video correlated positively with short version of Dental Anxiety Inventory (SDAxI) score (*n* = 20).

Brain regions	MNI coordinates			*z* value	Cluster size^a^	Voxel *P*^b^
	*x*	*y*	*z*			
*Scaler with sound*						
Insula R	39	−10	16	3.17	21	0.001
Supramarginal gyrus L	−3	50	37	3.24	16	0.001
*Turbine with sound*						
Insula L (1st peak)	−39	−13	−5	3.21	41	0.001
Insula L (2nd peak)	−36	14	7	2.93	14	0.002
Frontal operculum R	51	11	10	3.12	14	0.001
Frontal operculum L	−51	8	4	2.89	13	0.002

*^a^Cluster size is the number of voxels inside the cluster. ^b^Voxel P is the P value of the peak voxel at uncorrected P < 0.005 and cluster size >10 threshold. R, right; L, left*.

In addition, response to the scaler video in the left supramarginal gyrus positively correlated with the subject SDAxI score (Table 2). Response to the turbine video in the bilateral frontal operculum positively correlated with the subject SDAxI score (see Table 2). We did not find any significant negative correlation between brain responses to each video and subject SDAxI scores.

### Correlation Between Brain Activities and Dental Fear VAS Scores

The main focus of this study was the correlation between brain activities and SDAxI score. The correlation between brain activities and dental fear VAS score was reported here to support the findings from correlational analyses of SDAxI scores. For the scaler video, there was a positive correlation between activity in the right insula/frontal operculum and the corresponding dental fear VAS score (*P* = 0.001, Table 3). For the turbine video, there was a positive correlation between activity in the left insula/frontal operculum and the corresponding dental fear VAS score (*P* = 0.001, Table 3). We did not find any significant negative correlation between brain responses to each video and the corresponding dental fear VAS scores. Table 4 shows the values of VAS and brain activity for scaler and turbine videos, in descending order of VAS values for each video.

**Table 3:** Brain regions with level of activity in response to scaler or turbine video correlated positively with respective dental fear visual analog scale (VAS) score (*n* = 20).

Brain regions	MNI coordinates			*z* value	Cluster size^a^	Voxel *P*^b^
	*x*	*y*	*z*			
*Scaler with sound*						
Insula/Frontal operculum R	45	23	−2	3.26	44	0.001
Inferior frontal gyrus R	42	32	13	3.07	18	0.001
*Turbine with sound*						
Insula/Frontal operculum L	−45	17	7	3.01	23	0.001

*^a^Cluster size is the number of voxels inside the cluster. ^b^Voxel P is the P value of the peak voxel at uncorrected P < 0.005 and cluster size >10 threshold. R, right; L, left*.

**Table 4:** Brain activity level in response to scaler or turbine video with respective dental fear visual analog scale (VAS) score (*n* = 20).

	Scaler with sound		Turbine with sound
VAS (mm)	Effect size of brain activity in the right insula (MNI coordinate: 45, 23, −2)	VAS (mm)	Effect size of brain activity in the left insula (MNI coordinate: −45, 17, 7)
90	0.10	90	0.08
85	0.20	80	0.16
83	0.25	77	0.10
61	−0.08	75	0.11
50	−0.01	68	0.28
40	0.05	60	0.13
33	−0.08	56	0.11
30	−0.01	35	0.07
30	−0.07	30	0.00
30	0.14	30	0.17
30	0.12	28	−0.04
28	−0.03	25	−0.22
26	−0.03	25	−0.12
25	0.04	25	−0.04
22*	−0.08*	19	−0.24
22	0.06	15	−0.17
15	−0.01	10	−0.39
10	−0.12	5	−0.01
5	−0.09	4*	0.01*
0	−0.36	1	0.00

**Figure 3:**
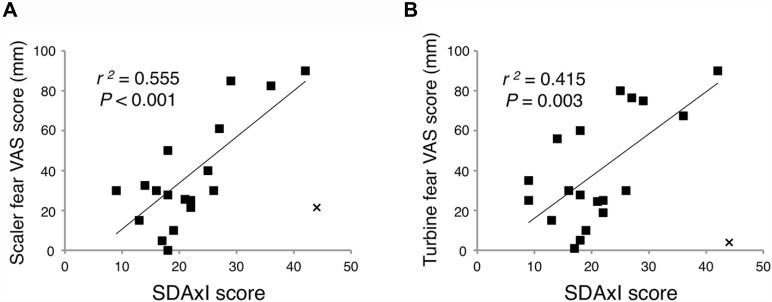
Associations of short version of Dental Anxiety Inventory (SDAxI) score with dental fear visual analog scale (VAS) scores of scaler or turbine video. **(A)** SDAxI and scaler VAS scores were positively correlated (*n* = 19).** (B)** SDAxI and turbine VAS scores were positively correlated (*n* = 19). For both correlations, we excluded one outlier that corresponded to the same participant (data point “x”). Both data had Cook’s distances more than three standard deviations from the mean Cook’s distance and were thus excluded.

VAS rounded to the nearest unit. Effect size rounded to tow decimal places. *Data from the subject considered as an outlier in Figure 3.

### Correlation Between SDAxI Score and Dental Fear VAS Scores

There was a positive correlation between SDAxI score and dental fear VAS score of the scaler (*r^2^* = 0.555, *P* < 0.001, Figure 3A) and of the turbine (*r*^2^ = 0.415, *P* = 0.003, Figure 3B), respectively. In the correlational analyses, Cook’s distance score was used to exclude outlier data points (Cook, 1977, 1979). This score for a given datum indicates how much the correlation will change by removing the datum. We defined an outlier as having a Cook’s distance score deviating more than three standard deviations from the mean. This is a commonly employed criterion for excluding outliers in correlational analyses (Sadeh et al., 2008, 2010; Rosso et al., 2010). As a result, we excluded one outlier from each correlation test, which belonged to the same participant.

## Discussion

### Summary of Major Findings

The novelty of the current study was the significant correlation between SDAxI score and insula response under stimuli mimicking dental treatment. The former assesses trait dental anxiety whereas the latter reflects state dental anxiety mimicking dental therapy. According to the cut-off thresholds by Ng and Leung (2008) set for SDAxI scores, the 20 individuals involved in the current study were mostly having average and high level of dental anxiety (45% each) while a minority had low anxiety level (10%). Ng and Leung (2008) revealed that the prevalence of low, average and high levels of dental anxiety were 9.6%, 79.9% and 10.5% respectively, in a 1,000 Chinese population, meaning within the current cohort, high trait dental anxiety individual to some extent seemed to be over-represented. We found that SDAxI score correlated positively with activities in the insula elicited by receiving audiovisual footages of scaler or turbine in action. As illustrated by Figure 3, participants with a higher SDAxI score had fMRI showing a higher level of activity in the insula and its adjacent frontal operculum when sensing audiovisual footages of either scaler or turbine (*r^2^* between 0.388 and 0.445). The results of this study confirmed our hypothesis that there is an association between the SDAxI score and the brain responses in the insula among healthy individuals upon exposure to stimuli with dental anxiety context. These pieces of evidence from brain data provided neurophysiological support showing SDAxI is an appropriate tool to evaluate trait dental anxiety level of patients who attend to dental practices.

### Dental Anxiety and the Brain Responses in the Insula

Trait anxiety describes a personality or characteristic of an individual (Chaplin et al., 1988; Endler and Kocovski, 2001) and SDAxI measures the dental trait anxiety of a person based on scenario type of questions (Stouthard et al., 1993; Ng et al., 2005). Our findings suggested that the dental anxiety state of an individual was neurologically encoded by the brain, in particularly the insula. The fact that there was an association between activities in the insula upon scaler or turbine video stimulus and subject based SDAxI score might imply some sort of hyper-reactivity in insular cortex in the specific regions reported might underpin the neurobiology of dental trait anxiety. This was consistent with Schienle et al. (2014) and Lin et al. (2015) in which a positive correlation was demonstrated between the responses in the insula triggered by silent dental treatment photographs with trait score of the State-Trait Anxiety Inventory or dental avoidance scores, respectively. While trait score of the State-Trait Anxiety Inventory is not specific for dental anxiety and dental avoidance score was generic and yet to be validated, results from the current study suggest that the standardized, commonly employed dental-specific SDAxI scale could be an effective tool in predicting dental anxiety biologically supported by corresponding neurophysiological evidence.

In the past, Schienle et al. (2014) found that activities in the insula did not correlate with Corah’s Dental Anxiety Scale score, an assessment scale for dental anxiety trait. Another of their studies also found that activities in the insula did not correlate with Corah’s Dental Anxiety Scale score unless the subjects were patients with dental phobia (Scharmüller et al., 2014). Regarding dental phobic patients, Hilbert et al. (2014) found that the activities in the insula did not correlate with Dental Fear Survey score, yet another trait dental anxiety specific scale. These inconsistencies can be partly attributed to the differences in stimuli employed and tasks given to the subjects. Stimuli could be silent, static dental treatment photographs (Scharmüller et al., 2014; Schienle et al., 2014) or combinations of different audio and video stimuli (Hilbert et al., 2014). Tasks involved in the correlational analyses could be focusing on distraction and perceived pain (Scharmüller et al., 2014; Schienle et al., 2014), or focusing on visual/auditory part of the stimuli (Hilbert et al., 2014). In this study, we employed audiovisual footages of operating dental scaler and turbine with matched sound to simulate dental treatment to be received by our subjects, and we instructed our subjects to pay attention to the stimulations as a whole without extra cognitive tasks. This time, we were able to reveal the association between activities in the insula and SDAxI score, a dental anxiety specific scale, among the healthy subjects. It has been reported that dental phobics had larger responses in a wide range of brain regions than non-phobics, such as in the prefrontal cortex, anterior cingulate cortex, insula, striatum, inferior parietal cortex, inferior occipital gyrus and middle temporal gyrus (Schienle et al., 2013; Scharmüller et al., 2014). This variety of brain regions involved may be partially explained by the use of slightly different exposures/stimuli to induce the dental anxiety in each cited study. Meanwhile, the current study is not totally innovative, but the importance of replicating some of the findings cannot be underemphasized.

From our results, it seemed that the correlation between SDAxI score (representing trait dental anxiety) and level of activity in the insular cortex triggered by audiovisual footages of scaler (representing state dental anxiety) only existed in the right insula, while that of turbine only existed in the left insula. The activations, or correlations, triggered by state anxiety due to exposure to dental, snake or spider pictures reported by previous studies were also sometimes located on the left hemisphere (Hilbert et al., 2014) and sometimes on the right (Straube et al., 2007; Lueken et al., 2011). Currently, we do not know why turbine affects the left insula more whereas scaler affects the right. The implication of this laterality remains to be elucidated.

The existing literature did not have neuroimaging data concerning the use of SDAxI or published meta-analyses reporting consistent activation pattern related to dental anxiety. To perform region-of-interest analyses instead of testing the whole brain, one needs to identify relevant region-of-interests either by literature review or from results of pilot studies. We did not have information from both sources. Nevertheless, we performed exploratory region-of-interest analyses within the insula, by pre-defining a mask that covered bilateral insula according to WFU PickAtlas toolbox incorporated in SPM 8. Based on the same initial statistical threshold, results identified the same clusters as for the whole brain analysis. After family-wise error (FWE) correction was considered, both voxel and cluster *P* > 0.05. Readers should be aware of this when the current results are interpreted.

It should be noted that the insula is heavily involved in affective processing, especially the processing of disgust (Wicker et al., 2003). No VAS rating on disgust was collected for participants in the current study. Future studies are needed to investigate whether disgust could be an underlying mechanism that partly explains dental anxiety and avoidance or a separate emotional processing that might confound the findings.

### SDAxI and Dental Care

Apart from correlating with activities in the insula, we also demonstrated a positive linear relationship between SDAxI scores and dental fear VAS scores of the scaler or turbine videos among the participants of the present study. This suggested that SDAxI was able to predict individually perceived dental fear level triggered by standard dental anxiety stimulus among the participants followed. Our participants aged between 18 and 61 and were comparable with the age range (25–64) from a previous community study (Ng and Leung, 2008). In the 1,000 participant community study, people with higher SDAxI scores had worse dental (higher decayed, missing and filled teeth index) and periodontal (more severe clinical attachment loss) conditions (Ng and Leung, 2008). The psychosocial-neurophysiological associations we found in this study is coherent with the notion that people with a higher SDAxI score are naturally more anxious while having their teeth fixed (drilling by turbine) or receiving treatment for gum disease (cleaning by scaler) underpinned by a certain neurobiological mechanism involving the insular cortex. Moreover, the lines of best fit in Figure 3 suggested that patients with trait dental anxiety up to SDAxI score >20 tended to have increased insular response upon mock state dental anxiety stimulation, which was consistent to the cut-off threshold of SDAxI score 21.3 reported by Ng and Leung (2008) confirming a tentative correlation between trait (SDAxI) and simulated state (VAS; neuro-response) dental anxiety. Putting both together, results indicated a higher neurological state dental anxiety in subject with higher trait dental anxiety. Clinicians may consider ways to relax patients with SDAxI score above this level, for anxiety control before commencing dental treatment, such as cognitive behavioral therapy (Getka and Glass, 1992), or providing more pre-operative information (Ng et al., 2004), or further evaluate the anxiety responses/reactions in previous dental experiences so as to consider any effective management in dental procedures.

### Limitations of the Study

This investigation, however, inherited own set of limitations due to its study design, mostly dealing with a small sample size and weak control of relative variables. For instance, a majority of the participants in the current study gave a low VAS rating to the audiovisual footage, implying that they might not have much state dental anxiety triggered. Meanwhile, the significant correlation between SDAxI and VAS might give us a more comprehensive picture by suggesting a possible connection between trait and state dental anxiety. Our convenient sample was relatively small with relatively high education background. It was reported that people with lower educational background were associated with a higher level of dental anxiety due to less accessibility to dental treatment (Moore et al., 1993). The sample was slightly heterogeneous with a few non-Chinese. A larger sample would better account for these inter-subject variations, especially that the current participants were all healthy, educated, and right-handed. For simplicity and time limitations, we neither tested simulations of other dental procedures nor asked follow-up questions that might further explore different aspects of dental experiences contributing to perceived anxiety. We did not measure the levels of anxiety of the participants in the scanner by means of real-time feedback or physiological data such as heart rate and/or galvanic skin response to prove if fear was provoked. Moreover, we could not really operate in patient’s mouth within the MRI machine, and the video footages were at best only surrogates of the real situation. Future studies can be extended to involve control subjects with no previous exposure to dentistry to see if SDAxI score could correlate with innate brain responses. The effect of level of cognition could also be examined by recruiting two cohorts with dental experience, one of small children and the other of matured adults, and compare their neuroimaging data and autonomic responses (Koda and Karibe, 2013; Karibe et al., 2014). All these should be taken into considerations when the results from this study were interpreted. The length of each trial in the current study lasted for approximately 14–20 s, corresponding to 0.05–0.07 Hz. Future studies may consider detecting brain signal in this frequency band specifically, as it may improve the signal to noise ratio of the data (Wang et al., 2014, 2015; Gao et al., 2018). Consequently, a more stringent statistical threshold (such as voxel-wise *P* < 0.05, FWE rate corrected) should be applied to further examine the associations. They should also consider assessment of perceived pain level during the stimulations, as both pain and dental anxiety involve the insula, and may exacerbate each other (van Wijk and Hoogstraten, 2009; van Wijk et al., 2010; Scharmüller et al., 2014).

## Conclusion

In conclusion, our results are the first to demonstrate an association between psychosocial measure of trait dental anxiety and the neurophysiological data among relatively higher educated healthy self-reported non-dental phobic individuals. There is a positive linear correlation between activity in the insula induced by sensing audiovisual footages of activated dental scaler or turbine and corresponding subject SDAxI scores.

## Data Availability

All datasets generated for this study are included in the manuscript.

## Ethics Statement

This study was carried out in accordance with the recommendations of The Institutional Review Board of The University of Hong Kong/Hospital Authority Hong Kong West Cluster with written informed consent from all subjects. All subjects gave written informed consent in accordance with the Declaration of Helsinki. The protocol was approved by The Institutional Review Board of The University of Hong Kong/Hospital Authority Hong Kong West Cluster.

## Author Contributions

All authors conceived the work and analyzed the data. JL and AY acquired the data and drafted the manuscript. HT, SN, P-LK, TG and WL critically revised the work. All authors have approved the final content of the manuscript.

## Conflict of Interest Statement

The authors declare that the research was conducted in the absence of any commercial or financial relationships that could be construed as a potential conflict of interest.

## References

[B1] Aartman I. H. (1998). Reliability and validity of the short version of the dental anxiety inventory. Community Dent. Oral Epidemiol. 26, 350–354. 10.1111/j.1600-0528.1998.tb01972.x 9792128

[B2] Armfield J. M. (2010a). Development and psychometric evaluation of the Index of Dental Anxiety and Fear (IDAF-4C^+^). Psychol. Assess. 22, 279–287. 10.1037/a0018678 20528055

[B3] Armfield J. M. (2010b). How do we measure dental fear and what are we measuring anyway. Oral Health Prev. Dent. 8, 107–115. 10.3290/j.ohpd.a19198 20589243

[B4] Burklund L. J. Eisenberger N. I. Lieberman M. D. (2007). The face of rejection: rejection sensitivity moderates dorsal anterior cingulate activity to disapproving facial expressions. Soc. Neurosci. 2, 238–253. 10.1080/17470910701391711 18461157 PMC2373282

[B5] Chaplin W. F. John O. P. Goldberg L. R. (1988). Conceptions of states and traits: dimensional attributes with ideals as prototypes. J. Pers. Soc. Psychol. 54, 541–557. 10.1037/0022-3514.54.4.541 3367279

[B6] Cook R. D. (1977). Detection of influential observation in linear regression. Technometrics 19, 15–18. 10.2307/1268249

[B7] Cook R. D. (1979). Influential observations in linear regression. J. Am. Stat. Assoc. 74, 169–174. 10.2307/2286747

[B8] de Jongh A. Olff M. van Hoolwerff H. Aartman I. H. Broekman B. Lindauer R.. (2008). Anxiety and post-traumatic stress symptoms following wisdom tooth removal. Behav. Res. Ther. 46, 1305–1310. 10.1016/j.brat.2008.09.004 18954863

[B9] de Jongh A. van Wijk A. J. Lindeboom J. A. (2011). Psychological impact of third molar surgery: a 1-month prospective study. J. Oral Maxillofac. Surg. 69, 59–65. 10.1016/j.joms.2010.05.073 20950915

[B10] Eisenberger N. I. Lieberman M. D. Williams K. D. (2003). Does rejection hurt? An fMRI study of social exclusion. Science 302, 290–292. 10.1126/science.1089134 14551436

[B11] Endler N. S. Kocovski N. L. (2001). State and trait anxiety revisited. J. Anxiety Disord. 15, 231–245. 10.1016/s0887-6185(01)00060-3 11442141

[B12] Forman S. D. Cohen J. D. Fitzgerald M. Eddy W. F. Mintun M. A. Noll D. C. (1995). Improved assessment of significant activation in functional magnetic resonance imaging (fMRI): use of a cluster-size threshold. Magn. Reson. Med. 33, 636–647. 10.1002/mrm.1910330508 7596267

[B13] Gale E. N. (1972). Fears of the dental situation. J. Dent. Res. 51, 964–966. 10.1177/00220345720510044001 4504718

[B14] Gao X. Gentile F. Rossion B. (2018). Fast periodic stimulation (FPS): a highly effective approach in fMRI brain mapping. Brain Struct. Funct. 223, 2433–2454. 10.1007/s00429-018-1630-4 29502144

[B15] Getka E. J. Glass C. R. (1992). Behavioral and cognitive-behavioral approaches to the reduction of dental anxiety. Behav. Ther. 23, 433–448. 10.1016/s0005-7894(05)80168-6

[B16] Goto T. K. Yeung A. W. K. Tanabe H. C. Ito Y. Jung H.-S. Ninomiya Y. (2016). Enhancement of combined umami and salty taste by glutathione in the human tongue and brain. Chem. Senses 41, 623–630. 10.1093/chemse/bjw066 27353260

[B17] Hilbert K. Evens R. Maslowski N. I. Wittchen H.-U. Lueken U. (2014). Fear processing in dental phobia during crossmodal symptom provocation: an fMRI study. Biomed. Res. Int. 2014:196353. 10.1155/2014/196353 24738049 PMC3967629

[B18] Ikeda N. Ayuse T. (2013). Reliability and validity of the short version of the dental anxiety inventory (SDAI) in a Japanese population. Acta Med. Nagasaki 58, 67–71.

[B19] Karibe H. Aoyagi-Naka K. Koda A. (2014). Maternal anxiety and child fear during dental procedures: a preliminary study. J. Dent. Child. 81, 72–77. 25198949

[B20] Koda A. Karibe H. (2013). Subjective ratings and autonomic responses to dental video stimulation in children and their mothers. Pediatr. Dent. J. 23, 79–85. 10.1016/j.pdj.2013.04.002

[B21] Leung W. K. Duan Y. R. Dong X. X. Yeung K. Zhou S. Y. Corbet E. F.. (2016). Perception of non-surgical periodontal treatment in individuals receiving or not receiving local anaesthesia. Oral Health Prev. Dent. 14, 165–175. 10.3290/j.ohpd.a35001 26525126

[B22] Lieberman M. D. Cunningham W. A. (2009). Type I and Type II error concerns in fMRI research: re-balancing the scale. Soc. Cogn. Affect. Neurosci. 4, 423–428. 10.1093/scan/nsp052 20035017 PMC2799956

[B23] Lin C.-S. Wu S.-Y. Wu L.-T. (2015). The anterior insula and anterior cingulate cortex are associated with avoidance of dental treatment based on prior experience of treatment in healthy adults. BMC Neurosci. 16:88. 10.1186/s12868-015-0224-9 26654201 PMC4676166

[B24] Lindeboom J. A. van Wijk A. J. (2010). A comparison of two implant techniques on patient-based outcome measures: a report of flapless vs. conventional flapped implant placement. Clin. Oral Implants Res. 21, 366–370. 10.1111/j.1600-0501.2009.01866.x 20128828

[B25] Lueken U. Kruschwitz J. D. Muehlhan M. Siegert J. Hoyer J. Wittchen H. U. (2011). How specific is specific phobia? Different neural response patterns in two subtypes of specific phobia. Neuroimage 56, 363–372. 10.1016/j.neuroimage.2011.02.015 21316468

[B26] McGrath C. Bedi R. (2004). The association between dental anxiety and oral health-related quality of life in Britain. Community Dent. Oral Epidemiol. 32, 67–72. 10.1111/j.1600-0528.2004.00119.x 14961842

[B27] McNeil D. W. Vrana S. R. Melamed B. G. Cuthbert B. N. Lang P. J. (1993). Emotional imagery in simple and social phobia: fear versus anxiety. J. Abnorm. Psychol. 102, 212–225. 10.1037/0021-843x.102.2.212 8315134

[B28] Moore R. Birn H. Kirkegaard E. Brødsgaard I. Scheutz F. (1993). Prevalence and characteristics of dental anxiety in Danish adults. Community Dent. Oral Epidemiol. 21, 292–296. 10.1111/j.1600-0528.1993.tb00777.x 8222604

[B29] Mumford J. A. Nichols T. E. (2008). Power calculation for group fMRI studies accounting for arbitrary design and temporal autocorrelation. Neuroimage 39, 261–268. 10.1016/j.neuroimage.2007.07.061 17919925 PMC2423281

[B31] Ng S. K. Chau A. W. Leung W. K. (2004). The effect of pre-operative information in relieving anxiety in oral surgery patients. Community Dent. Oral Epidemiol. 32, 227–235. 10.1111/j.1600-0528.2004.00161.x 15151693

[B30] Ng S. K. Leung W. K. (2008). A community study on the relationship of dental anxiety with oral health status and oral health-related quality of life. Community Dent. Oral Epidemiol. 36, 347–356. 10.1111/j.1600-0528.2007.00412.x 19145721

[B32] Ng S. K. Stouthard M. E. Leung W. (2005). Validation of a chinese version of the dental anxiety inventory. Community Dent. Oral Epidemiol. 33, 107–114. 10.1111/j.1600-0528.2004.00199.x 15725173

[B34] Oosterink F. de Jongh A. Aartman I. H. (2008). What are people afraid of during dental treatment? Anxiety-provoking capacity of 67 stimuli characteristic of the dental setting. Eur. J. Oral Sci. 116, 44–51. 10.1111/j.1600-0722.2007.00500.x 18186731

[B33] Oosterink F. de Jongh A. Aartman I. H. (2009). Negative events and their potential risk of precipitating pathological forms of dental anxiety. J. Anxiety Disord. 23, 451–457. 10.1016/j.janxdis.2008.09.002 18990543

[B35] Rosso I. M. Makris N. Britton J. C. Price L. M. Gold A. L. Zai D.. (2010). Anxiety sensitivity correlates with two indices of right anterior insula structure in specific animal phobia. Depress. Anxiety 27, 1104–1110. 10.1002/da.20765 21132846 PMC3010373

[B36] Sadeh B. Podlipsky I. Zhdanov A. Yovel G. (2010). Event-related potential and functional MRI measures of face-selectivity are highly correlated: a simultaneous ERP-fMRI investigation. Hum. Brain Mapp. 31, 1490–1501. 10.1002/hbm.20952 20127870 PMC6870976

[B37] Sadeh B. Zhdanov A. Podlipsky I. Hendler T. Yovel G. (2008). The validity of the face-selective ERP N170 component during simultaneous recording with functional MRI. Neuroimage 42, 778–786. 10.1016/j.neuroimage.2008.04.168 18554929

[B38] Scharmüller W. Übel S. Leutgeb V. Schoengassner F. Wabnegger A. Schienle A. (2014). Do not think about pain: neural correlates of attention guiding during visual symptom provocation in dental phobia—an fMRI study. Brain Res. 1566, 69–76. 10.1016/j.brainres.2014.04.017 24751570

[B39] Schienle A. Scharmüller W. Leutgeb V. Schäfer A. Stark R. (2013). Sex differences in the functional and structural neuroanatomy of dental phobia. Brain Struct. Funct. 218, 779–787. 10.1007/s00429-012-0428-z 22644919

[B40] Schienle A. Wabnegger A. Schoengassner F. Scharmüller W. (2014). Neuronal correlates of three attentional strategies during affective picture processing: an fMRI study. Cogn. Affect. Behav. Neurosci. 14, 1320–1326. 10.3758/s13415-014-0274-y 24710652

[B41] Stouthard M. Hoogstraten J. (1987). Ratings of fears associated with twelve dental situations. J. Dent. Res. 66, 1175–1178. 10.1177/00220345870660061601 3476589

[B42] Stouthard M. E. Hoogstraten J. Mellenbergh G. J. (1995). A study on the convergent and discriminant validity of the Dental Anxiety Inventory. Behav. Res. Ther. 33, 589–595. 10.1016/0005-7967(94)00096-3 7598683

[B43] Stouthard M. E. Mellenbergh G. J. Hoogstraten J. (1993). Assessment of dental anxiety: a facet approach. Anxiety Stress Coping 6, 89–105. 10.1080/10615809308248372

[B44] Straube T. Mentzel H.-J. Miltner W. H. (2007). Waiting for spiders: brain activation during anticipatory anxiety in spider phobics. Neuroimage 37, 1427–1436. 10.1016/j.neuroimage.2007.06.023 17681799

[B45] Strother S. C. (2006). Evaluating fMRI preprocessing pipelines. IEEE Eng. Med. Biol. Mag. 25, 27–41. 10.1109/memb.2006.1607667 16568935

[B46] Taylor J. Deane F. P. (2002). Development of a short form of the Test Anxiety Inventory (TAI). J. Gen. Psychol. 129, 127–136. 10.1080/00221300209603133 12153130

[B47] van Wijk A. J. Hoogstraten J. (2009). Anxiety and pain during dental injections. J. Dent. 37, 700–704. 10.1016/j.jdent.2009.05.023 19556053

[B48] van Wijk A. J. de Jongh A. Lindeboom J. A. (2010). Anxiety sensitivity as a predictor of anxiety and pain related to third molar removal. J. Oral Maxillofac. Surg. 68, 2723–2729. 10.1016/j.joms.2010.06.174 20833462

[B49] Vermaire J. H. de Jongh A. Aartman I. H. A. (2008). Dental anxiety and quality of life: the effect of dental treatment. Community Dent. Oral Epidemiol. 36, 409–416. 10.1111/j.1600-0528.2007.00416.x 18924257

[B50] Vlaeyen J. W. Kole-Snijders A. M. Boeren R. G. van Eek H. (1995). Fear of movement/(re) injury in chronic low back pain and its relation to behavioral performance. Pain 62, 363–372. 10.1016/0304-3959(94)00279-n 8657437

[B51] Wang Y.-F. Dai G.-S. Liu F. Long Z.-L. Yan J. H. Chen H.-F. (2015). Steady-state BOLD response to higher-order cognition modulates low-frequency neural oscillations. J. Cogn. Neurosci. 27, 2406–2415. 10.1162/jocn_a_00864 26284992

[B52] Wang Y.-F. Liu F. Long Z.-L. Duan X.-J. Cui Q. Yan J. H.. (2014). Steady-state BOLD response modulates low frequency neural oscillations. Sci. Rep. 4:7376. 10.1038/srep07376 25488025 PMC4260215

[B53] Wicker B. Keysers C. Plailly J. Royet J.-P. Gallese V. Rizzolatti G. (2003). Both of us disgusted in my insula: the common neural basis of seeing and feeling disgust. Neuron 40, 655–664. 10.1016/S0896-6273(03)00679-2 14642287

[B54] Wong H. M. Mak C. M. To W. M. (2015). Development of a dental anxiety provoking scale: a pilot study in Hong Kong. J. Dent. Sci. 10, 240–247. 10.1016/j.jds.2014.09.003

[B55] Wong H. M. Mak C. M. Xu Y. F. (2011). A four-part setting on examining the anxiety-provoking capacity of the sound of dental equipment. Noise Health 13, 385–391. 10.4103/1463-1741.90291 22122954

[B56] Worsley K. (2001). “Statistical analysis of activation images,” in Functional MRI: An Introduction to Methods, eds Jezzard P. Matthews P. M. Smith S. M. (Oxford: Oxford University Press), 251–270.

[B57] Yeung A. W. K. Goto T. K. Leung W. K. (2017). A bibliometric review of research trends in neuroimaging. Curr. Sci. 112, 725–734. 10.18520/cs/v112/i04/725-734

[B58] Yeung A. W. K. Goto T. K. Leung W. K. (2019). Brain responses to stimuli mimicking dental treatment among non-phobic individuals: a meta-analysis. Oral Dis. 25, 34–43. 10.1111/odi.12819 29250913

[B59] Yeung A. W. K. Tanabe H. C. Suen J. L. K. Goto T. K. (2016). Taste intensity modulates effective connectivity from the insular cortex to the thalamus in humans. Neuroimage 135, 214–222. 10.1016/j.neuroimage.2016.04.057 27132544

